# 90-day mortality risk related to postoperative potassium levels in patients undergoing coronary bypass surgery

**DOI:** 10.1016/j.jmccpl.2023.100035

**Published:** 2023-05-02

**Authors:** Mikkel Kjeldgaard, Mads Odgaard Mæng, Christian Torp-Pedersen, Peter Søgaard, Kristian Hay Kragholm, Jan Jesper Andreasen, Maria Lukács Krogager

**Affiliations:** a Unit of Epidemiology and Biostatistics, Aalborg University Hospital, Søndre Skovvej 15, 9000 Aalborg, Denmark; b Faculty of Health Science, Aalborg University, Fredrik Bajers Vej 5, 9100 Aalborg, Denmark; c Department of Cardiology, Aalborg University Hospital, Hobrovej 18-22, 9000 Aalborg, Denmark; d Department of Cardiology and Clinical Research, Nordsjællands Hospital, Kongens Vænge 2, 3400 Hillerød, Denmark; e Department of Cardiothoracic Surgery, Aalborg University Hospital, Hobrovej 18-22, 9000 Aalborg, Denmark; f Department of Clinical Medicine, Aalborg University, Søndre Skovvej 15, 9000 Aalborg, Denmark

**Keywords:** Coronary artery bypass grafting, Potassium, Electrolyte disturbance, Short-term mortality

## Abstract

**Aims:**

While electrolyte depletion is known to occur during coronary artery bypass grafting (CABG) with extracorporeal circulation, little is known about the frequency of potassium disturbances following either on- or off-pump CABG and its association with mortality. We examined the frequency of potassium disturbances and the association of plasma potassium with mortality risk in patients following CABG.

**Methods and results:**

From Danish National Registries, we identified 6123 adult patients (≥18 years old) undergoing first-time CABG, and who had a registered potassium measurement within 14 days before and 7 days after their surgery between 1995 and 2018. Using 4.0–4.6 mmol/L as reference, potassium was stratified into five predefined intervals: <3.5, 3.5–3.9, 4.0–4.6, 4.7–5.0, and ≥5.1 mmol/L. We examined the absolute mortality risk and assessed the Cox proportional hazard model to analyze the 90-day all-cause mortality risk in relation to the first available post-operative potassium sample. Pre- and postoperative potassium disturbances were rare, while more common in patients with chronic kidney disease. The adjusted cox regression presented a trend of increased mortality only in hyperkalemia. The absolute mortality risk increased in hyperkalemia, hypokalemia and low-normokalemia, while high normokalemia presented a lesser relative risk of mortality, compared to the reference of 4.0–4.6 mmol/L.

**Conclusion:**

Although the cox regression presented a trend of increased mortality only in hyperkalemia, the absolute mortality risk supported a strategy of careful monitoring and evaluation of any potassium disturbance, including in the lower normokalemia interval.

## Highlights

• Potassium disturbances following coronary artery bypass grafting (CABG) and its association with mortality
• Danish, nationwide register study including 6.123 patients undergoing first-time CABG
• Pre- and postoperative potassium disturbances were rare.
• High-normokalemia had a lower mortality risk than other potassium levels.
• This study emphasizes careful monitoring of pre- and postoperative potassium levels in patients undergoing CABG.

## Graphical abstract

**Figure:**
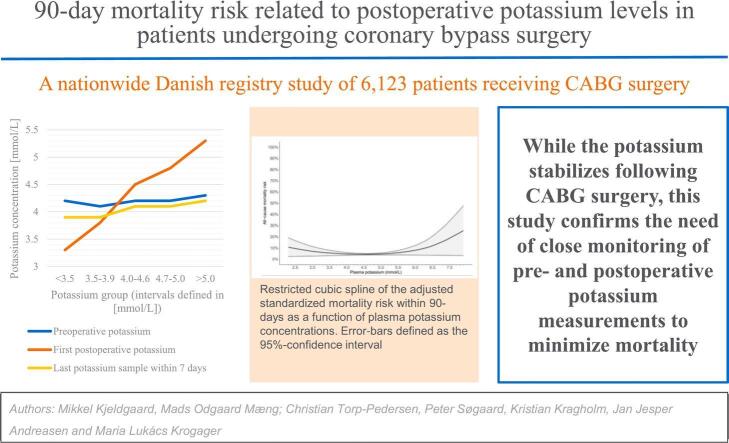

## 1 Introduction

Electrolyte depletions are common during coronary artery bypass grafting (CABG) with extracorporeal circulation, despite electrolyte supplements throughout the procedure [1]. The underlying mechanism appears to be a combination extracorporeal circulation, hypothermia-induced diuresis, and intracellular shifts of electrolytes during surgery [1], [2], [3].

Among patients undergoing CABG, other cardiovascular diseases, such as hypertension and heart failure, are common comorbidities associated with potassium disturbances and hereby increased mortality risk [1], [2], [4], [5], [6].

Studies about potassium disturbances in different populations with cardiovascular disease have shown a U-shaped association of potassium concentrations and mortality. These studies also found an association of increased mortality within the high and low levels of the normal potassium range [6], [7], [8], [9], [10].

CABG, the underlying disease and pharmacological therapy all represent a risk for developing potassium disturbances. Nonetheless, little is known about the frequency of potassium disturbances following CABG and its association with mortality. Especially, whether potassium within normal range also represent a risk in patients undergoing cardiac surgery.

To address this issue, we used data from 6123 patients undergoing either on- or off-pump coronary artery bypass grafting (CABG) to examine an association between different plasma potassium concentrations and all-cause mortality.

## 2 Methods

### 2.1 Databases

Data was collected through the national Danish registries using linkage of unique civil registration numbers throughout the registries.

### 2.2 Study population

The study population was defined by individuals aged 18 or older assigned with the Classification of Surgical Procedures (NCSP) codes regarding patients receiving first-time CABG surgery in the period of 1995–2018.

A potassium sample within 14 days prior and seven days following surgery was required for inclusion. To ensure best quality data and ease of presenting the results, we collected only plasma potassium measurements [7], [11], [12], [13].

### 2.3 Comorbidities, procedures, and concomitant medications

The Danish National Patient Register was used to identify patients with previous history of ischemic heart disease (IHD), previous or recent myocardial infarction, atrial flutter (AFLU) or -fibrillation (AFIB), heart failure (HF), stroke, arterial hypertension, previous acute kidney disease (AKD), chronic kidney disease, chronic need of dialysis, diabetes mellitus, chronic obstructive pulmonary disease (COPD), chronic liver disease (CLD), inflammatory bowel disease (IBD), and peripheral artery disease (PAD). Supplementary material S1 Table, S2 Table, and S3 Table through their different ICD-, procedure-, and ATC-codes.

Hypertension and diabetes mellitus were defined by ICD-10 or through the redemption of at least two relevant drugs in two concomitant quarters before the surgery date and seven days after surgery date [11], [12].

The estimated glomerular filtration rate (eGFR) was calculated using serum creatinine concentrations [14]. Hereby, the kidney function before and after the surgery were accessed. Renal insufficiency was defined by an eGFR <30 mL/min/1.73m^2^.

Patients diagnosed with syndrome of inappropriate antidiuretic hormone secretion, Addison's Disease, hyperaldosteronism, and diabetes insipidus within five years prior to the surgery date were excluded from this study due to their association with abnormal potassium levels.

The Danish National Prescription Register was used to identify prescriptions of acetylsalicylic acid, agents acting on the renin-angiotensin system, antidiabetics, beta blocking agents, beta-2 agonists, corticoids, digitalis glycosides (digoxin), non-steroid anti-inflammatory, potassium supplements, vasopressin and analogues, antihypertensive drug divisions, and diuretic drug divisions (S1, S2, and S3 Tables in Supplementary material).

The study population selection is presented in Supplementary material S4 as a flow-chart with illustration of inclusion and exclusion criteria.

### 2.4 Outcome

The primary outcome of the study was all-cause mortality within 90 days following CABG.

### 2.5 Statistical analysis

Categorical variables were reported as counts and percentages and continuous variables as medians with 25th and 75th percentiles. Differences between variables were compared using χ2 and Kruskal Wallis tests, as appropriate.

Using a restricted cubic spline model, the mortality risk as a continuous hazard function of plasma potassium was illustrated. Knots were set at the 10th, 50th and 90th percentiles of the plasma potassium concentrations. The reference interval and the different strata for survival analyses were selected by a combined evaluation of the restricted cubic spline model, the interval with the lowest mortality and by the interval with approximately most patients in the group [13]. The intervals can be seen in Table 1.

**Table 1:** Stratified plasma potassium intervals and classification.

Potassium interval [mmol/L]	Classification
<3.5	Hypokalemia
3.5–3.9	Low normal-range potassium
4.0–4.6	Reference
4.7–5.0	High normal-range potassium
≥5.1	Hyperkalemia

Kaplan-Meier cumulative mortality curves were constructed for each of the plasma potassium strata to illustrate survival probability.

We investigated the association of the postoperative plasma potassium concentrations and the mortality within 90 days using Cox proportional hazard model. Selected through clinical experience, the following comorbidities were chosen for the multivariable model: age, gender, renal insufficiency, hypertension, heart failure, potassium supplements, beta2-agonist, and digoxin.

The cox proportional hazards model assumptions were examined, and no linearity of mortality and age were found. Through quantile cut-off examination at 20 %, 40 %, 60 % and 80 %, age was stratified for the multivariable analysis into: 18–58, 59–65, 66–70, 71–74, and >75 years as intervals with 18–50 years as reference.

Using a cox regression, the absolute mortality risk was examined in an age, gender, comorbidity, and drug standardized population, with potassium 4.0–4.6 mmol/L as reference.

To test the robustness of the main results, five sensitivity analyses were conducted, and the selected subgroups were defined as: no diagnosis of kidney failure, no diagnosis of cancer, patients with a preoperative‑potassium within the normal-range, the last potassium sample during hospitalization and the last potassium sample within seven days of surgery.

Hazard ratios (HR) and absolute risks (AR) were estimated using 95 % confidence intervals (95 % CI). All data management and analyses were performed by using SAS 9.4 (SAS Institute Inc., Cary, NC, USA) and R Statistical Software version 3.5.0 (R Development Core Team) [15].

## 3 Results

### 3.1 Patients and characteristics

The characteristics of the study population are presented in Table 2. The study population were stratified according to predefined potassium intervals of the first post-operative potassium measurement, see Table 1.

**Table 2:** Patient characteristics according to each group based on stratification of the first post-operative potassium. Data are represented as mean ± SD (age), median (IQR), or number of patients and percentages. Ischemic heart disease (IHD), myocardial infarction (MI), atrial flutter (AFLU), atrial fibrillation (AFIB), acute kidney disease (AKD), chronic obstructive pulmonary disease (COPD), chronic kidney disease (CKD), chronic liver disease (CLD), inflammatory bowel disease (IBD), mineralocorticoid receptor antagonist (MRA), Angiotensin converting enzyme-inhibitor (ACEI), angiotensin II receptor blocker (ARB), non-steroidal anti-inflammatory drug (NSAID). Data with four or less observations were noted as ≤4 in regard of protection of personal data. (n) denotes the total count of patients present in each group. The p-value of significance were calculated using Chi-square test for categorical variables and Kruskal-Wallis test for non-parametric variables.

Potassium group (mmol/L (N))		<3.5 (n = 37)	3.5–3.9 (n = 232)	4.0–4.6 (n = 2960)	4.7–5.0 (n = 2018)	≥5.1 (n = 876)	Total (n = 6123)	p-Value
Age	Median (range)	68 (44, 82)	71 (29, 86)	68 (30, 95)	68 (31, 87)	70 (31, 88)	68 (29, 95)	<0.0001
Gender	Female	11 (29.7)	62 (26.7)	550 (18.6)	392 (19.4)	150 (17.1)	1165 (19.0)	
	Male	26 (70.3)	170 (73.3)	2410 (81.4)	1626 (80.6)	726 (82.9)	4958 (81.0)	0.00633
Pre-operative potassium	Median (range)	4.2 (2.9, 4.9)	4.1 (3.1, 5.3)	4.2 (2.1, 5.8)	4.2 (3.0, 6.1)	4.3 (3.0, 6.2)	4.2 (2.1, 6.2)	<0.0001
Pre-operative potassium grouped	<3.5 mmol/L	≤4	13	71	26	5	≤119	
	3.5–4.5 mmol/L	31 (83.8)	208 (89.7)	2603 (87.9)	1757 (87.1)	691 (78.9)	5290 (86.4)	
	>4.5 mmol/L	≤4	11	286	235	180	≤716	<0.0001
Post-surgery potassium	Median (range)	3.3 (2.3, 3.4)	3.8 (3.5, 3.9)	4.5 (4.0, 4.6)	4.8 (4.7, 5.0)	5.3 (5.1, 7.4)	4.6 (2.3, 7.4)	<0.0001
Last potassium within 7 days	Median (range)	3.9 (2.9, 4.9)	3.9 (2.6, 5.5)	4.1 (2.5, 12.1)	4.1 (2.6, 6.1)	4.2 (2.7, 6.4)	4.1 (2.5, 12.1)	<0.0001
Last potassium during submission	<3.5 mmol/L	7 (18.9)	18 (7.8)	183 (6.2)	112 (5.6)	42 (4.8)	362 (5.9)	
	3.5–4.5 mmol/L	27 (73.0)	195 (84.1)	2453 (82.9)	1635 (81.0)	668 (76.3)	4978 (81.3)	
	>4.5 mmol/L	≤4	19	324	271	166	≤784	<0.0001
Days to normalized potassium	Median (range)	5 (1, 28)	9 (1, 29)	1 (1, 30)	1 (1, 7)	1 (1, 6)	1 (1, 30)	<0.0001
pre-operative sodium	Median (range)	139 (122, 144)	140 (129, 149)	141 (123, 151)	141 (122, 149)	140 (121, 156)	141 (121, 156)	<0.0001
Pre-operative creatinine	Median (range)	93 (51, 914)	90 (43, 657)	89 (32, 964)	90 (41, 963)	96 (40, 1020)	90 (32, 1020)	<0.0001
Post-operative sodium	Median (range)	140 (127, 153)	139 (120, 155)	138 (125, 154)	137 (125, 155)	137 (126, 150)	138 (120, 155)	<0.0001
Post-operative creatinine	Median (range)	90 (51, 914)	91 (46, 657)	89 (37, 1140)	90 (41, 963)	97 (41, 1050)	90 (37, 1140)	<0.0001
Time from surgery to first potassium sample	Median (range)	2 (1, 7)	2 (1, 7)	1 (1, 7)	1 (1, 7)	1 (1, 6)	1 (1, 7)	<0.0001
Comorbidities								
IHD/MI	Number (%)	36 (97.3)	228 (98.3)	2945 (99.5)	2006 (99.4)	869 (99.2)	6084 (99.4)	0.0901
AFLU/AFIB	Number (%)	9 (24.3)	49 (21.1)	520 (17.6)	371 (18.4)	197 (22.5)	1146 (18.7)	0.0137
Heart failure	Number (%)	0	≤4	14	12	8	≤38	
Stroke	Number (%)	≤4	26	218	151	77	≤476	
Hypertension	Number (%)	16 (43.2)	96 (41.4)	1153 (39.0)	812 (40.2)	318 (36.3)	2395 (39.1)	0.311
Previous AKD		10 (27.0)	51 (22.0)	548 (18.5)	394 (19.5)	275 (31.4)	1278 (20.9)	<0.0001
Chronic dialysis need	Number (%)	≤4	7	93	55	25	≤184	
Diabetes mellitus	Number (%)	19 (51.4)	110 (47.4)	1250 (42.2)	785 (38.9)	391 (44.6)	2555 (41.7)	0.00591
COPD	Number (%)	6 (16.2)	23 (9.9)	285 (9.6)	185 (9.2)	94 (10.7)	593 (9.7)	0.472
CKD	Number (%)	7 (18.9)	32 (13.8)	232 (7.8)	130 (6.4)	147 (16.8)	548 (8.9)	<0.0001
CLD	Number (%)	0	≤4	30	8	7	≤49	
IBD	Number (%)	≤4	≤4	20	10	8	≤46	
MI within 30 days	Number (%)	≤4	24	314	239	118	≤699	
PAD	Number (%)	10 (27.0)	52 (22.4)	451 (15.2)	288 (14.3)	139 (15.9)	940 (15.4)	0.00512
Pharmacotherapy								
Diuretics	Number (%)	10 (27.0)	56 (24.1)	645 (21.8)	438 (21.7)	215 (24.5)	1364 (22.3)	0.363
Thiazide	Number (%)	5 (13.5)	23 (9.9)	227 (7.7)	163 (8.1)	73 (8.3)	491 (8.0)	0.515
Sulfonamide	Number (%)	0	0	≤4	8	≤4	≤16	
Loop	Number (%)	5 (13.5)	33 (14.2)	416 (14.1)	267 (13.2)	140 (16.0)	861 (14.1)	0.428
MRA	Number (%)	0 (0.0)	6 (2.6)	74 (2.5)	54 (2.7)	34 (3.9)	168 (2.7)	0.199
Potassium-sparing	Number (%)	5 (13.5)	22 (9.5)	230 (7.8)	170 (8.4)	75 (8.6)	502 (8.2)	0.573
ACEI & ARB	Number (%)	17 (45.9)	122 (52.6)	1384 (46.8)	976 (48.4)	456 (52.1)	2955 (48.3)	0.0488
Beta-blocker	Number (%)	25 (67.6)	132 (56.9)	1838 (62.1)	1343 (66.6)	536 (61.2)	3874 (63.3)	0.00186
Potassium-supplement	Number (%)	10 (27.0)	70 (30.2)	785 (26.5)	538 (26.7)	266 (30.4)	1669 (27.3)	0.169
Digoxin	Number (%)	10 (27.0)	67 (28.9)	774 (26.1)	536 (26.6)	260 (29.7)	1647 (26.9)	0.300
NSAID	Number (%)	10 (27.0)	75 (32.3)	917 (31.0)	621 (30.8)	310 (35.4)	1933 (31.6)	0.116
Beta-2-agonist	Number (%)	12 (32.4)	64 (27.6)	820 (27.7)	566 (28.0)	273 (31.2)	1735 (28.3)	0.344
Corticoids	Number (%)	10 (27.0)	64 (27.6)	751 (25.4)	516 (25.6)	255 (29.1)	1596 (26.1)	0.239
Diseased within 90-days	Number (%)	≤4	14	114	69	48	≤249	

Overall, we observed a median preoperative potassium at 4.2 mmol/L [IQR: 2.1–6.2] and a postoperative potassium concentration of 4.6 mmol/L [IQR: 2.3–7.4].

Hypokalemia represented ≤1.9 % of the preoperative- and 0.60 % of the postoperative potassium samples. In comparison, hyperkalemia represented 11.69 % prior to surgery and 14.31 % following surgery.

**Fig. 1:**
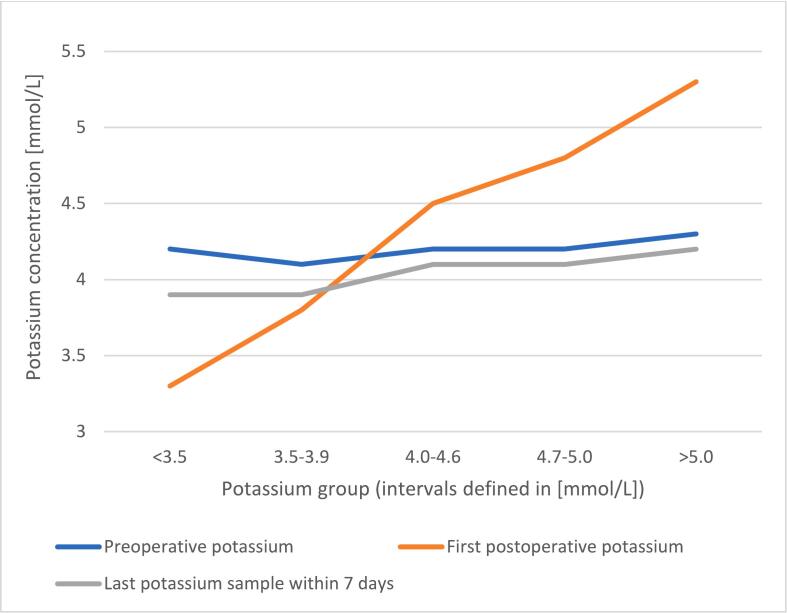
Preoperative, postoperative, and last-available potassium sample within 7 days, according to each group (defined by stratification of the postoperative potassium concentration). Total count of patients (n) = 6123. The patients were stratified according to the first post-operative potassium and counted: <3.5 (n = 37); 3.5–3.9 (n = 232); 4.0–4.6 (n = 2960); 4.7–5.0 (n = 2018); and ≥5.1 (n = 876).

Fig. 1 illustrates the preoperative, postoperative, and last available potassium samples within 7 days, for each study group (stratified by the first-postoperative potassium sample). Table 2 reveals that the preoperative and last available potassium samples were similar, regardless of the postoperative potassium sample, as illustrated in Fig. 1.

Chronic kidney disease was more frequent with potassium disturbances where 18.9 % had hypokalemia, 13.8 % low-normokalemia, and 16.8 % with hyperkalemia. Compared to 7.8 % and 6.4 % of the reference interval and high-normokalemia, respectively.

### 3.2 Survival analysis

**Fig. 2:**
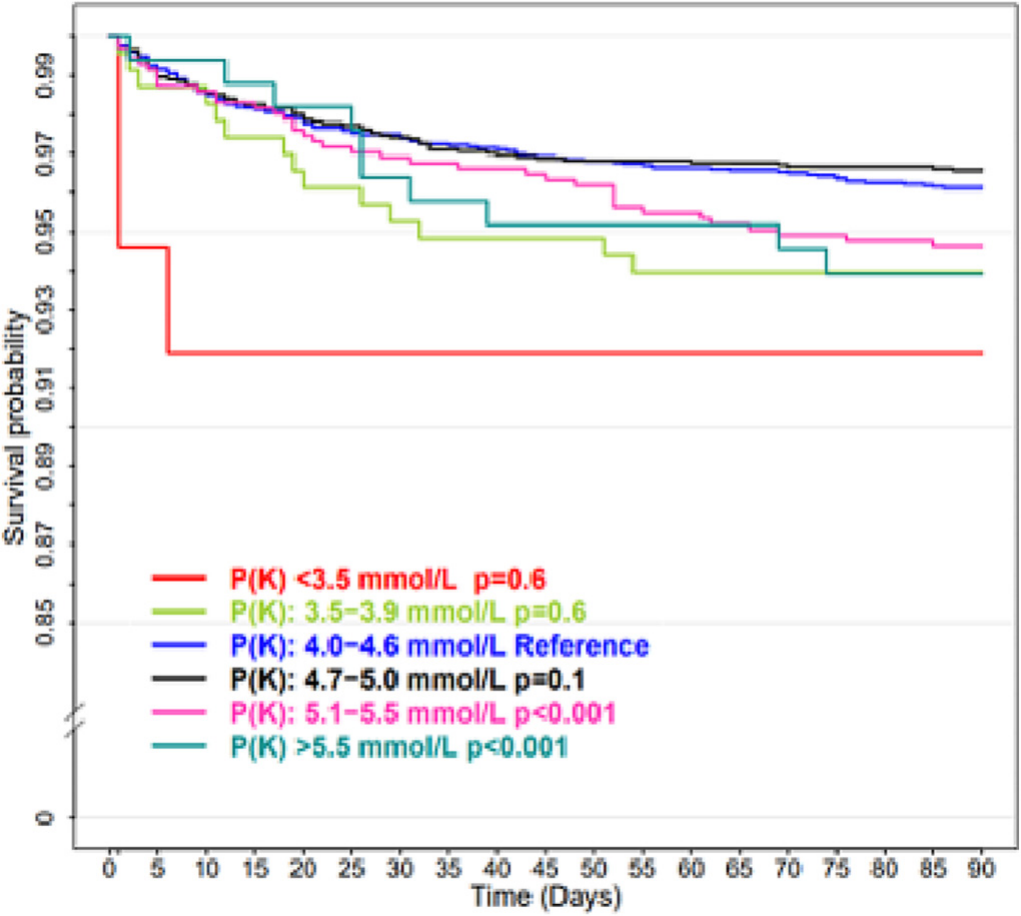
Kaplan-Meier analysis of the survival probability among the different potassium intervals. Total count of patients (n) = 6123. The patients were stratified according to the first post-operative potassium and counted: <3.5 (n = 37); 3.5–3.9 (n = 232); 4.0–4.6 (n = 2960); 4.7–5.0 (n = 2018); and ≥5.1 (n = 876).

Within 90-days, mortality rates in the predefined potassium intervals (defined by stratification of the postoperative potassium) from the lowest (K: <3.5 mmol/L) to the highest (K: >5.5 mmol/L) were ≤10.81, 6.03, 3.85, 3.42 and 5.48 % respectively as illustrated in the Kaplan-Meier survival curves in Fig. 2 and in Table 2.

**Fig. 3:**
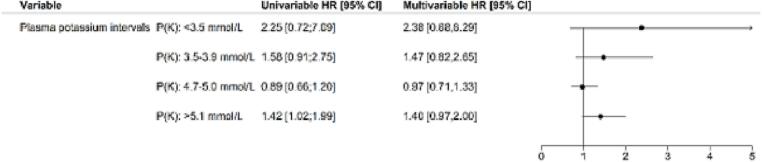
All-cause mortality using Cox-proportional hazards regression analysis in patients who have underwent a CABG stratified by potassium intervals (90-day-follow-up). Model adjusted for covariates. Reference interval represented by the interval K: 4.0–4.6 mmol/L. Significance was defined as 95 %-confidence interval, i.e. p-value < 0.05. Total count of patients (n) = 6123. The patients were stratified according to the first post-operative potassium and counted: <3.5 (n = 37); 3.5–3.9 (n = 232); 4.0–4.6 (n = 2960); 4.7–5.0 (n = 2018); and ≥5.1 (n = 876).

Compared with the reference potassium strata of 4.0–4.6 mmol/L, the univariable Cox regression analysis presented a significant increased mortality risk in patients with potassium concentrations >5.1 mmol/L ((hazard ratio) HR 1.42; 95 % confidence interval (CI), 1.02–1.99). However, when adjusting for relevant covariables, the p-value did not remain significant (HR 1.4; 95 % CI, 0.97–2.00, Fig. 3).

The absolute risk of mortality was presented in Table 3 and was adjusted for age, gender, renal insufficiency, hypertension, heart failure, potassium supplements, beta2-agonist, and digoxin. With 4.0–4.6 mmol/L as reference, the absolute risk of mortality (AR) increased in the strata of potassium ≥5.1 mmol/L ((AR) 0.049), at low-normokalemia ((AR) 0.051), and at hypokalemia ((AR) 0.071) compared to the reference with an (AR) of 0.039. High-normokalemia presented a just slightly lower AR of 0.038.

**Table 3:** Absolute risk of 90-day mortality adjusted for age, gender, renal insufficiency, hypertension, heart failure, potassium supplements, beta2-agonist, and digoxin. (Relative risk = variable risk / reference risk.) (Difference in risk = reference risk − variable risk.) Total count of patients (n) = 6123. The patients were stratified according to the first post-operative potassium and counted: <3.5 (n = 37); 3.5–3.9 (n = 232); 4.0–4.6 (n = 2960); 4.7–5.0 (n = 2018); and ≥ 5.1 (n = 876).

Potassium strata	Unadjusted model			Adjusted model		
	Absolute risk	Relative risk	Risk difference	Absolute risk	Relative risk	Risk difference
<3.5 mmol/L	0.033	0.83	−0.0069	0.071	1.82	−0.032
3.5–3.9 mmol/L	0.048	1.2	0.0080	0.051	1.31	−0.012
4.0–4.6 mmol/L	0.040	Reference		0.039	Reference	
4.7.5.0 mmol/L	0.92	−0.92	0.0031	0.038	0.98	0.00076
≥5.1 mmol/L	1.2	−12	−0.0082	0.049	1.26	−0.010

**Fig. 4:**
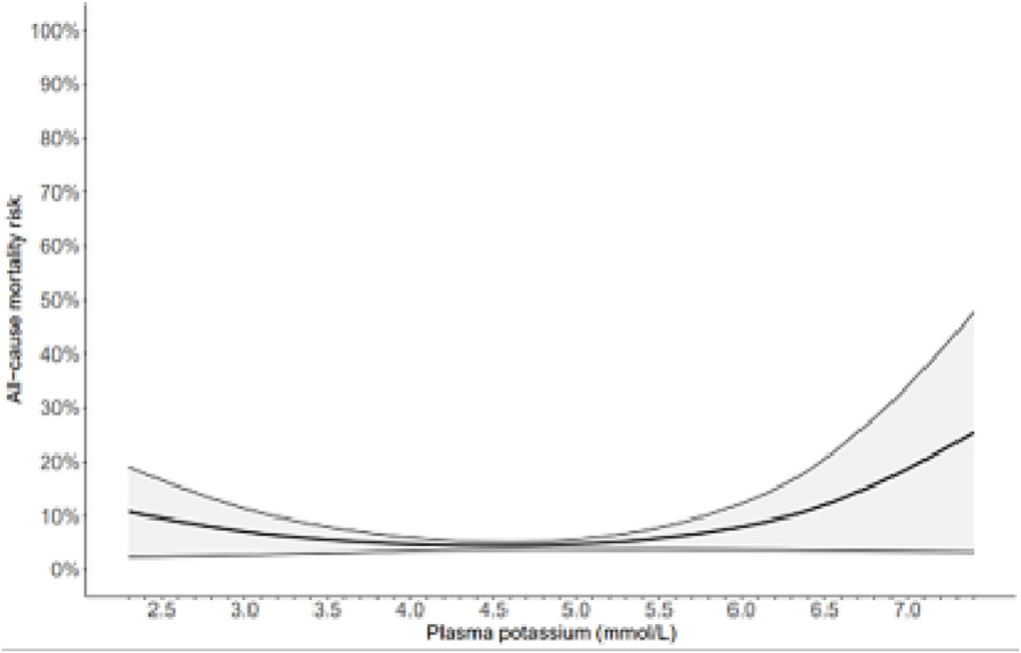
Restricted cubic spline of the adjusted standardized mortality risk within 90-days as a function of plasma potassium concentrations. Error-bars defined as the 95 %-confidence interval. Knots at 10th, 50th and 90th percentiles of potassium. Model adjusted for age, gender, renal insufficiency, hypertension, heart failure, and prescriptions for beta-2 agonists, digoxin, and potassium supplements. Total count of patients (n) = 6123. The patients were stratified according to the first post-operative potassium and counted: <3.5 (n = 37); 3.5–3.9 (n = 232); 4.0–4.6 (n = 2960); 4.7–5.0 (n = 2018); and ≥5.1 (n = 876). This figure shows an approximation of the function relating plasma potassium to the hazard rate of death and should not be interpreted with respect to some reference.

The standardized risk of mortality within 90-days was presented in Fig. 4. The spline curve showed high mortality risk for both hypokalemia and hyperkalemia. In addition, the spline curve illustrated increased risk of death in patients with potassium concentrations outside the interval 4.2–5.2 mmol/L.

### 3.3 Additional analysis

Five sensitivity analyses were conducted to investigate the presence of factors interfering with the overall survival in the population. The results of these analysis were presented in Supplementary material S.5–7 Figures for further data.

The first analysis of patients with no kidney-failure (eGFR>30) presented similar results as the main analysis.

The results remained similar to the main analysis when excluding patients with a cancer-diagnosis within five years prior to surgery.

Third, when comparing the last available potassium sample during submission, potassium concentrations >4.6 mmol/L were associated with increased 90-day mortality risk.

The fourth analysis, only comparing patients with a normal pre-operative potassium, and the fifth analysis, comparing the last available potassium sample within seven days, found similar results as the main analysis.

## 4 Discussion

This study included 6123 patients undergoing first-time CABG and analyzed short-term mortality risk in relation to different potassium intervals in patients undergoing first-time CABG.

The study had four major findings:

(i) Pre- and postoperative potassium disturbances were rare.
(ii) The adjusted cox regression only presented a trend of increased mortality risk in post-operative hyperkalemia.
(iii) The absolute mortality risk assessment found an increased risk ratio in hyperkalemia, hypokalemia and low-normokalemia (3.5–3.9 mmol/L), while presenting a lesser relative risk in high-normokalemia (4.7–5.0 mmol/L).
(iv) The standardized mortality risk curve illustrated an increased all-cause mortality for potassium samples outside potassium interval 4.2–5.2 mmol/L.

An association of abnormal potassium concentrations with increased mortality risk was expected [6], [7], [8], [16], [17].

Interestingly, this study found no significant associations in the Cox regression. The results might be influenced by the low count of observations in hypokalemia compared to the other strata, which might affect the significance estimations of the results.

However, with 4.0–4.6 mmol/L as reference, the absolute mortality-risk model did not only present a risk increase in hyper- and hypokalemia, but also in low-normokalemia and a lesser relative risk in high-normokalemia. Therefore, the results from the present study confirm that careful attention must be paid to the patients' plasma potassium concentrations in the early postoperative period.

Following surgery there were fewer cases of postoperative hypokalemia, while there was an increased tendency of hyperkalemia. Potassium disturbances could be expected in the days following surgery due to extravascular fluctuations and intensive potassium supplementation during cardiac surgery with extracorporeal circulation [1], [2], [18].

This imbalance may explain an increased risk of tachyarrhythmia and mortality, which was observed in the assessment of absolute risk of mortality [1], [19], [20].

Another study found post-operative atrial arrhythmias among 67 %, 47 %, and 50 % of patients with plasma potassium measurements of respectively 2.5–2. mmol/L, 3.0–3.4 mmol/L, and 5.5–6.0 mmol/L, compared to 36 % of the reference group (4.5–4.9 mmol/L) [21].

We performed five sensitivity analyses to test the robustness of the main analysis.

It was interesting that the sensitivity analysis excluding patients with a previous cancer diagnosis presented no difference in mortality. This was expected because patients with cancer are in increased risk of malnutritional- and metabolic disorders due to the tumor or its treatment, this study found no difference [3], [22]. This suggests the patients either had a mild cancer, were successfully treated, or the cancer did not affect metabolic derangements.

### 4.1 Study limitations

It was possible to obtain unique, reliable data from the Danish National Health registries determining comorbid diseases and concomitant medication, along with potassium measurements and date of death, which strengthen the reliability of our findings. Every factor considered as a possible confounder was included in the Cox multivariable analysis.

The specific cause of death was not available in this study. Therefore, this study defined a limited follow-up time, from which the cause of death may be related to the cardiac illness and procedure.

It was not possible to obtain information on whether the CABG procedures were performed as off-pump procedures averting extracorporeal circulation. Yet, CABG surgeries are usually performed on-pump [23], [24]. Around 25 % of CABG surgeries were performed off-pump in 2016 [24], [25]. However, as early, and late survival rates for both off-pump and on-pump CABC are similar [24]. we do not expect that the missing information on whether on- or off-pump surgery was used have any impact on the results from the present study [23].

Intraoperative data including urine output, potassium drawn during surgery and medication during the surgery as well as hospital-based medications were not available.

Danish National Health registries do not contain diagnoses from the general practitioner. For this reason, we determined comorbid illnesses as hypertension and diabetes mellitus by using an alternative method based on diagnoses codes and/or two or more prescriptions of concomitant medications that may increase the predictive value. Withal, misclassification cannot be excluded.

Although our study presented a 39.1 % incidence rate of arterial hypertension among the study population, which could be considered as a relatively low incidence, we defined hypertension as redemption of minimum two antihypertensive drugs in two concomitant quarters. This definition of hypertension was previously validated by Olesen et al. [26] Hospital-based ICD-10 coding for hypertension is not as sensitive, as most of the patients treated for high blood pressure do not require hospitalization or an ambulatory contact where this code is registered.

Further, this study reported a high frequency of diabetes mellitus among patients receiving coronary artery bypass grafting (CABG) surgery. This finding has been consistently reported in the literature, with studies showing that 25–30 % of all patients undergoing CABG have diabetes, and up to 60 % of patients with acute myocardial infarction have previously undiagnosed diabetes, according to Armstrong et al. [27] Additionally, Goel et al. reported that during a 5-year follow-up, CABG had significantly lower rates of myocardial infarctions and death compared to percutaneous coronary intervention (PCI) treatment, which could contribute to the high incidence of diabetes mellitus among patients undergoing CABG [28].

Despite adjusting for several variables and performing five sensitivity analyses to test the robustness of the main results, we were not able to adjust for all known confounders. We did not *e.g.*, adjust for the per-operative use of allogeneic red blood cell transfusion, which have been shown to be associated with increased mortality during cardiac surgery [29].

Furthermore, unknown confounders must also always be considered possible.

Finally, it is not possible to confirm whether mortality is increased due to the effect of potassium influence on myocardial membrane, or it is an indicative of other mechanisms.

### 4.2 Recommendations by the authors

Our study results suggest that patients with potassium disturbances in the early postoperative period have a higher short-term mortality risk compared to those with normokalemia. We believe that potassium disturbances can be perceived both as a risk factor and a risk marker of disease. In some cases, potassium disturbances could have led to arrhythmia development, in other cases they can reflect post-surgical complications such as kidney insufficiency or infections.

## 5 Conclusion

In this register-based study of 6123 patients undergoing first-time CABG between 1995 and 2018, pre- and postoperative potassium disturbances were rare.

The absolute mortality risk was increased in hyperkalemia, hypokalemia and low-normokalemia, while high-normokalemia presented a lesser relative risk of mortality, compared to the reference of 4.0–4.6 mmol/L.

## Clinical implications

Although the cox regression presented a trend of increased mortality only in hyperkalemia, the absolute mortality risk was increased in hypokalemia and in the low-normokalemia strata of 3.5–3.9 mmol/L. The high-normokalemia 4.7–5.0 presented a lesser risk relative to the reference strata of 4.0–4.6 mmol/L.

To prevent any relative risk increase, this study thus confirms the need of careful monitoring and evaluation of the pre- and postoperative potassium samples in patients undergoing CABG.

## Funding

Bachelor research grant from 10.13039/501100002702Aalborg University, Aalborg, Denmark.

## Declaration of competing interest

The authors report no relationships that could be construed as a conflict of interest.

All authors take responsibility for all aspects of the reliability and freedom from bias of the data presented and their discussed interpretation.

## Appendix A Supplementary data

Supplementary material Image 1
