# Supplementary material for: 90-day mortality risk related to postoperative potassium levels in patients undergoing coronary bypass surgery
Source: J Mol Cell Cardiol Plus. 2023 May 2;4:100035. doi: 10.1016/j.jmccpl.2023.100035 (PMC11708319; doi:10.1016/j.jmccpl.2023.100035)
Supplement: Supplementary file 1 — Supplementary material [file mmc1.docx]

SUPPLEMENTAL MATERIAL

S1. Table Definitions of comorbidities, procedures, and concomitant medications based on different ICD-10, NOMESCO Classification of Surgical Procedures (NCSP), and ATC codes identified prior to index date.

|  | **ICD-10 codes** | **Time**  **prior to index date** | **NCSP**  **codes** | **Time**  **prior to index date** | **ATC codes** | **Time**  **prior to index date** |
| --- | --- | --- | --- | --- | --- | --- |
| **Comorbidities and procedures** | | | | | | |
| Coronary artery bypass graft | − | − | KFNA,  KFNB,  KFNC,  KFND,  KFNE | − | − | − |
| Valve replacement | − | − | KFG,  KFK,  KFM, KFJ | − | − | − |
| Implantable cardioverter defibrillator | − | − | BCFB0, BCF00-03 | 5 years | − | − |
| Hemodialysis | − | − | BJFD20 | 5 years | − | − |
| Percutaneous coronary intervention |  | − | KFNG | 5 years | − | − |
| Atrial flutter or fibrillation | I48 | 5 years | − | − | − | − |
| Cancer | C00-99 | 5 years | − | − | − | − |
| Chronic kidney disease | I120,  Z992,  N391,  N02-8,  N11-14, | 5 years | − | − | − | − |

|  | N16, N18-  19, N158159, Q61,  M321B, |  |  |  |  |  |
| --- | --- | --- | --- | --- | --- | --- |
| Chronic liver disease | B18, C22, K71-77 | 5 years | − | − | − | − |
| Chronic obstructive pulmonary disease | J40-44 | 5 years | − | − | − | − |
| Diabetes | E10-14 | 5 years | − | − | A10 | 180 days |
| Heart failure | I110, I130,  I132, I42, I50, J81 | 5 years | − | − | − | − |
| Hypertension | DI11-15 | 5 years | − | − | − | − |
| Ischemic heart disease including myocardial infarction | I20-25 | 5 years |  |  | − | − |
| Peripheral artery disease | DI70  DI71  DI72  DI73  DI74  DI77  DI78  DI79 DI652A | 5 years | − | − | − | − |
| Stroke | DI61,  DI62,  DI63,  DI64,  DG458-  459,  DG433-  438 | 5 years | − | − | − | − |
| Inflammatory bowel disease (IBD) | DK50-51 | 5 years | − | − | − | − |
| **Concomitant medications** | |  |  |  |  |  |
| Acetylsalicylic acid | − | − | − | − | N02BA01, | 180 days |
| Agents acting on the renin-angiotensin  system | − | − | − | − | C09 | 180 days |
| Antidiabetics | − | − | − | − | A10 | 180 days |
| Beta blocking agents | − | − | − | − | C07 | 180 days |
| Beta-2 agonists | − | − | − | − | R03C | 180 days |
| Digitalis glycosides | − | − | − | − | C01AA | 180 days |
| Antimicrobials | − | − | − | − | A07AA | 180 days |
| Loop diuretics | − | − | − | − | C03C | 180 days |
| Non-steroid antiinflammatory and antirheumatic products | − | − | − | − | M01A | 180 days |
| Potassium supplements | − | − | − | − | A12B | 180 days |

S2. Table List of antihypertensive drugs and corresponding ATC codes used to define hypertension

| **ATC code** | **Name of drug** |
| --- | --- |
| C02A | Antiadrenergic agents, centrally acting |
| C02B | Antiadrenergic agents, ganglion blocking |
| C02C | Antiadrenergic agents, peripherally acting |
| C02DA | Thiazide-derivatives |
| C02DB | Hydrazynophthalazin-derivatives |
| C02DD | Nitroferricyanide-derivatives |
| C02DG | Guanidin-derivatives |
| C02L | Antihypertensives and diuretics in combination |
| C03AA | Thiazides |
| C03AB | Thiazides and potassium in combination |
| C03BA | Sulfonamides |
| C03BB | Sulfonamides and potassium in combination |
| C03C | Loop diuretics |
| C03DA | Aldosteron antagonists |
| C03DB | Other potassium sparing agents |
| C03EA | Low-ceiling diuretics and potassium sparing agents |
| C03EB | High-ceiling diuretics and potassium sparing agents |
| C03X | Other diuretics |
| C07A | Beta-blockers |
| C07B | Beta-blockers and thiazides |
| C07C | Beta-blockers and other diuretics |
| C07D | Beta-blockers, thiazides and other diuretics |
| C07FB | Beta-blockers and calcium antagonists |
| C07FX | Beta-blockers and other combinations |
| C08C | Selective calcium antagonists primarily with vascular effect |
| C08D | Selective calcium antagonists with direct cardiac effect |
| C08E | Non-selective calcium antagonists |
| C08G | Calcium antagonists and diuretics |
| C09AA | Angiotensin converting enzyme inhibitors |
| C09BA | Angiotensin converting enzyme inhibitors and diuretics |
| C09BB | Angiotensin converting enzyme inhibitors and calcium antagonists |
| C09CA | Angiotensin II antagonists |
| C09DA | Angiotensin II antagonists and diuretics |

S3. Table List of drugs and corresponding Anatomical Therapeutic Chemical System (ATC) codes used for identifying diuretics

| **ATC code** | **Name of drug** |
| --- | --- |
| C03AA | Thiazides |
| C03AB | Thiazides and potassium in combination |
| C03BA | Sulfonamides |
| C03BB | Sulfonamides and potassium in combination |
| C03CA | Loop diuretics |
| C03CB | Loop diuretics and potassium in combination |
| C03DA | Aldosteron antagonists |
| C03DB | Other potassium sparing agents |
| C03EA | Low-ceiling diuretics and potassium sparing agents |
| C03EB | High-ceiling diuretics and potassium sparing agents |
| C03X | Other diuretics |

S4. Population flow-chart


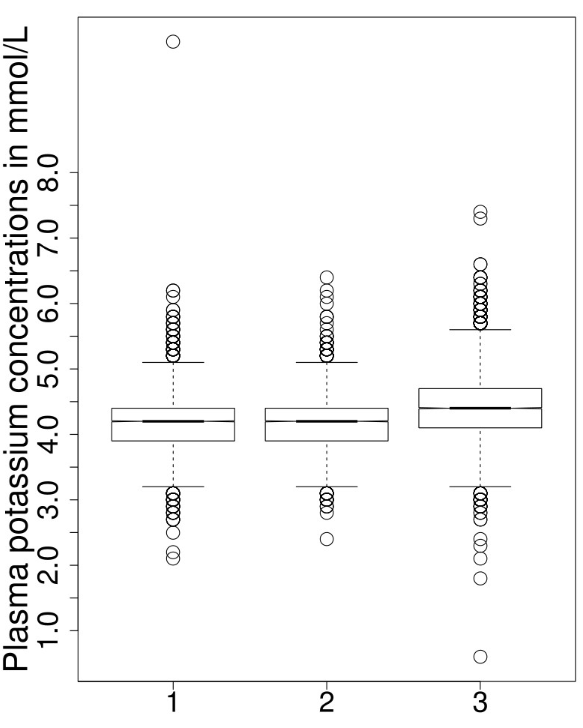
S5. Boxplot of the plasma potassium groups taken within 1-7 days after surgery

The plasma potassium concentrations for preoperative potassium (1), postoperative potassium (2), and finally the average of all potassium samples taken within 1-7 days after surgery (3).

S6. Histogram of the time from open heart surgery to the first potassium measurement.


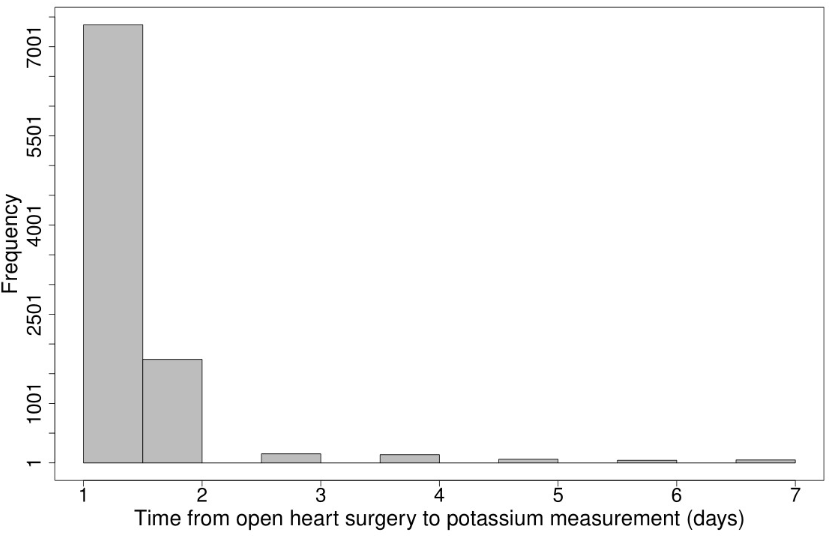


S7. Table of sensitivity analyses

|  | **Univariable model** | | | **Multivariable model** | | |
| --- | --- | --- | --- | --- | --- | --- |
|  | **HR** | **95% CI** | **p-value** | **HR** | **95% CI** | **p-value** |
| **No acute kidney disease (N= 8356)** | | | | | | |
| <3.5 mmol/L | 0.79 | [0.25;2.48] | 0.6855 | 0.70 | [0.22;2.23] | 0.55 |
| 3.5-3.9 mmol/L | 0.90 | [0.56;1.43] | 0.6445 | 0.82 | [0.51;1.30] | 0.39 |
| 4.0-4.6 mmol/L | Reference | | | | | |
| 4.7.5.0 mmol/L | 1.37 | [0.99;1.89] | 0.0589 | 1.45 | [1.05;2.01] | 0.026 |
| 5.1-5.5 mmol/L | 1.95 | [1.26;3.01] | 0.0026 | 1.97 | [1.27;3.06] | 0.0026 |
| >5.5 mmol/L | 3.25 | [1.58;6.66] | 0.0013 | 3.55 | [1.71;7.36] | < 0.001 |
| **No past history of malignancy (N=9626)** | | | | | | |
| <3.5 mmol/L | 1.35 | [0.67;2.75] | 0.4041 | 1.00 | [0.49;2.05] | 1.0 |
| 3.5-3.9 mmol/L | 0.94 | [0.65;1.36] | 0.7473 | 0.82 | [0.56;1.18] | 0.29 |
| 4.7.5.0 mmol/L | 1.25 | [0.96;1.63] | 0.0981 | 1.31 | [1.00;1.71] | 0.049 |
| 5.1-5.5 mmol/L | 2.03 | [1.47;2.82] | <0.001 | 1.78 | [1.28;2.49] | <0.001 |
| >5.5 mmol/L | 2.96 | [1.77;4.95] | <0.001 | 2.61 | [1.54;4.41] | <0.001 |
| **CABG only (N=8768)** | | | | | | |
| <3.5 mmol/L | 1.34 | [0.55;3.28] | 0.5201 | 1.17 | [0.48;2.88] | 0.73 |
| 3.5-3.9 mmol/L | 0.92 | [0.59;1.45] | 0.7275 | 0.83 | [0.53;1.31] | 0.42 |
| 4.7.5.0 mmol/L | 1.25 | [0.92;1.70] | 0.1491 | 1.22 | [0.89;1.66] | 0.21 |
| 5.1-5.5 mmol/L | 2.30 | [1.61;3.28] | <0.001 | 1.87 | [1.29;2.69] | <0.001 |
| >5.5 mmol/L | 2.22 | [1.13;4.36] | 0.0212 | 1.68 | [0.84;3.35] | 0.14 |
| **Normokalemia preoperatively (n=9062)** | | | | | | |
| <3.5 mmol/L | 1.49 | [0.70;3.18] | 0.3016 | 1.20 | [0.55;2.60] | 0.65 |
| 3.5-3.9 mmol/L | 0.88 | [0.59;1.32] | 0.5455 | 0.77 | [0.52;1.15] | 0.20 |
| 4.7.5.0 mmol/L | 1.30 | [0.98;1.72] | 0.0655 | 1.34 | [1.01;1.78] | 0.044 |
| 5.1-5.5 mmol/L | 2.18 | [1.54;3.09] | <0.001 | 1.91 | [1.34;2.72] | < 0.001 |
| >5.5 mmol/L | 3.36 | [1.90;5.92] | <0.001 | 3.18 | [1.79;5.66] | < 0.001 |
| **Last available potassium measurement within 1-7 days postoperatively** | | | | | | |
| <3.5 mmol/L | 1.39 | [0.95;2.02] | 0.08876 | 1.02 | [0.69;1.49] | 0.93 |
| 3.5-3.9 mmol/L | 0.64 | [0.49;0.85] | 0.00164 | 0.60 | [0.46;0.80] | < 0.001 |
| 4.7.5.0 mmol/L | 3.23 | [2.42;4.30] | < 0.001 | 3.19 | [2.39;4.27] | < 0.001 |
| 5.1-5.5 mmol/L | 8.43 | [6.02;11.80] | < 0.001 | 6.15 | [4.37;8.65] | < 0.001 |
| >5.5 mmol/L | 19.38 | [12.02;31.25] | < 0.001 | 12.30 | [7.51;20.13] | < 0.001 |
